# The Epigenetic Battleground: Host Chromatin at the Core of Infection

**DOI:** 10.3390/epigenomes10010013

**Published:** 2026-02-15

**Authors:** Fabrício Castro Machado, Nilmar Silvio Moretti

**Affiliations:** Laboratório de Biologia Molecular de Patógenos (LBMP), Departamento de Microbiologia, Imunologia e Parasitologia, Escola Paulista de Medicina, Universidade Federal de São Paulo—UNIFESP, São Paulo 04039-032, Brazil; fcmachado@unifesp.br

**Keywords:** chromatin, epigenetic, host and pathogen interaction, acetylation, methylation, transcriptional regulation, immune system, genome dynamics

## Abstract

Chromatin dynamics are usually modulated by histone epigenetic post-translational modifications, which rapidly and reversibly govern accessibility and transcriptional responsiveness. During microbial infection, this regulatory layer becomes a highly contested interface where host defense mechanisms and pathogen-driven subversion strategies converge and compete. Many infectious agents exploit chromatin to reprogram gene expression, creating cellular environments that are conducive to infection, proliferation, and persistence. Diverse strategies have been described for viruses, bacteria, fungi, protozoa and nematodes, including the direct secretion of acetyltransferases and methyltransferases, interference with host chromatin-binding proteins, subcellular localization of transcriptional factors or epigenetic regulators, and metabolic availability manipulation. Concurrently, host cells activate immune and stress-response genes to mount rapid, adaptable antimicrobial responses. Recent advances in genome-wide, single-cell, and spatial omics profiling have begun to reveal the temporal and cell-type-specific dynamics of the host genome at the core of infection. This review synthesizes current insights into how chromatin is rewired by the major categories of pathogens during infection, highlighting representative case studies across infective agents and the functional consequences for immunity and cell fate. In addition, we discuss emerging techniques for epigenomic and transcriptomic data collection, and the potential of targeted host-directed therapeutic strategies. Chromatin regulation is thus a promising field of study and a possible target for next-generation interventions.

## 1. Introduction: Underlying Mechanisms for Interaction with Pathogens

The human genome encodes only ~20,000 genes. However, proteome complexity depends less on the gene number per se than it does on recombination, transcription and translation regulation, splicing, and especially epigenetic control, which regulates access to genetic information [1,2,3]. Human genes are multiexonic, with an average of around seven splice isoforms, which expand coding potential early in gene expression [4]. Cellular responsiveness is further amplified by post-translational modifications (PTMs), which generate distinct proteoforms [5]. This results in a cellular proteome comprising millions of distinct molecular entities [6]. More than 600 distinct PTM types have been described to date [7,8]. These are introduced co-translationally or post-translationally [3,9] to control structure, stability, binding, enzymatic activity and trafficking [10]. PTMs often function in coordinated and hierarchical combinations, a phenomenon known as PTM crosstalk [11], which provides cellular homeostasis while also creating potential interfaces for pathogen subversion.

Central to epigenetic control is chromatin, in which DNA is packaged into nucleosomes composed of ~147 base pairs of DNA wrapped around an octamer of histone proteins. Histone PTMs are usually written at N-terminal tails, where they modulate chromatin structure and accessibility for transcription [12]. One of the most studied PTMs is acetylation, which targets ε-amino groups of lysine residues, neutralizing their positive charge and altering protein interactions [13,14]. The biochemical foundation of this PTM, such as the discovery of coenzyme A and its link to cellular metabolism, were recognized with Nobel Prizes in 1953 and 1954 [15]. Direct evidence was described in early 1960s via acetylated residues found in histones that were isolated from calf thymus cells [16].

Histone PTM levels can be reversibly catalyzed and are enzymatically controlled by the opposing actions of PTM “writer” and “eraser” proteins, which enzymatically add or remove modifications, respectively. “Reader” proteins with specialized domains recognize PTMs and recruit downstream effector complexes [17,18]. Writers include histone acetyltransferases (HATs) and histone methyltransferases (HMTs), which deposit acetyl and methyl marks on histones and non-histone proteins [19]. Erasers include- histone deacetylases (HDACs) and histone demethylases (HDMs), which remove these modifications and modulate transcriptional outcomes [20]. Within this architecture, the dynamic balance between transcriptionally permissive euchromatin and repressive heterochromatin, long known to be altered by histone acetylation and methylation [21], reflects the intimate interdependence between PTMs and gene-regulatory pathways. This is because chromatin DNA–histone complexes constitute the responsive substrate that orchestrates virtually all transcriptional cellular programs, from development to immunity [22]. All these discoveries have laid the foundation for the current dogma that states that levels of histone modifications directly contribute to the regulation of gene transcription.

At the molecular level, nucleosomes are the fundamental units of structural chromatin and contain dozens of modifiable residues. These epigenetic PTM states define the so-called “histone code” [23]. Genome-wide studies have shown that lysines such as H3K4, H3K9, H3K27, H3K36, and H4K20 exhibit multiple methylation states. Alternatively, residues including H3K9, H3K14, H3K18, and H3K27 undergo highly dynamic acetylation–deacetylation cycles, integrating developmental, metabolic, and inflammatory cues into transcriptional outcomes [24]. Consistent patterns emerge from this code: H3K4me1 and H3K4me3 are strongly associated with transcriptional activation [25], whereas H3K27me3, together with H3K9me2/3, marks transcriptionally repressed chromatin domains [26,27]. Other layers of chromatin control consist of DNA methylation, recruitment of chromatin-remodeling complexes, and long non-coding RNAs [28]. Overall, chromatin represents an attractive target for pathogens because it sits at the convergence of transcriptional control and cellular signal integration, allowing relatively limited perturbations to generate broad, durable, and coordinated effects on host gene expression.

Importantly, chromatin modulation during infection is not limited to local histone modifications but operates within regulatory frameworks that include higher-order genome organization and RNA-mediated control [29]. The genome is three-dimensionally structured with chromatin loops, topologically associating domains (TADs), and compartments that define or constrain regulatory interactions of transcriptional neighborhoods. Spatial architecture perturbation during infection, especially in association with viruses, offers these pathogens an efficient means of reprogramming host gene expression [30]. Recent Hi-C-based studies have revealed that viral infections, such as SARS-CoV-2, can induce compartment switching and disrupt long-range chromatin interactions at immune gene loci, resulting in attenuated antiviral responses and global transcriptional rewiring [31]. In parallel, non-coding RNAs (ncRNAs) further extend these regulatory tools by acting as intermediaries between infection-induced signaling and chromatin modification, in different classes of hosts, from plants to animals [32,33,34]. Micro RNAs (miRNAs) and long non-coding RNAs (lncRNAs) are frequently reprogrammed during infection and can influence chromatin states by regulating chromatin-modifying enzymes or by directly recruiting these complexes to defined genomic regions. Overall, regulation by 3D genome organization and ncRNA can complement mechanisms based on histone PTMs, underscoring the multi-layered nature of chromatin, which serves as a strategic target during infection.

In addition to chromatin and histones, PTMs such as acetylation play key regulatory roles in non-histone proteins. For example, the transcription factor NF-κB is regulated by lysine acetylation, which modulates its translocation to the nucleus, as well as its transcriptional activity and inflammatory signaling output [35]. Generally, cells can dynamically regulate genome accessibility via reversible PTMs, enabling precise control of gene expression in homeostasis, as well as in response to environmental challenges and pathogens.

In immune cells, rapid gene induction via the removal of a repressive methyl mark or the addition of an acetyl group to lysine residues in histones and non-histones can typically increase chromatin accessibility and facilitate the swift transcription of immune-related genes. This epigenetic flexibility enables the production of inflammatory cytokines, chemokines, and antimicrobial proteins required for effective host defense [36]. Notably, several pathogens have evolved mechanisms to epigenetically manipulate host chromatin regulation, especially histone PTMs, as key targets in host–pathogen interactions [37,38].

There is a competitive interaction for epigenetic control between hosts and pathogens, which occurs at the level of host chromatin. Among the complex molecular mechanisms in this battleground, directly or indirectly interfering with transcription greatly mediates the genetic code expression by acting as on/off switches for cellular pathways and behavior outcomes. This review provides paradigmatic case studies and a comprehensive overview of how pathogens interact with the host chromatin and transcription during infection, from invasion to proliferation and dissemination. This highlights the emerging and multifaceted roles of transcriptional control and histone PTMs, such as acetylation and methylation, as pivotal mediators in this host–pathogen relationship.

## 2. Chromatin Under Attack: How Pathogens Exploit Genome Dynamics

Pathogens entering the host cell do not encounter a simple and passive genome, but a protected and highly structured chromatin environment in which epigenetic marks act as flags and boundaries to control gene expression. During infection, chromatin is further regulated in the nucleus, with epigenetic patterns shifting within minutes to rapidly reshape cellular and immune responses. Thus, host–pathogen interaction is extended beyond the competition for resources and becomes a direct struggle for survival by controlling the transcriptional landscape and limiting the magnitude of host defenses.

The ability of pathogens to manipulate host nuclear processes was first demonstrated in plant systems by phytopathogens. In the late 1970s, studies of *Agrobacterium tumefaciens* revealed that this bacterium genetically transforms plant cells by integrating its tumor-inducing (Ti) plasmid into the host genome, driving uncontrolled cell proliferation and the production of metabolites that support bacterial survival [39]. This discovery provided early evidence that pathogens can directly target host nuclear functions to promote infection.

Efficient communication between the nucleus and cytoplasm is one of the essential biological processes for proper gene expression, with the movement of materials between the nucleus and cytoplasm (known as nucleocytoplasmic exchange) being crucial for both transcription and translation. Chromatin-binding proteins, such as histones, “writers”, “readers”, “erasers”, and transcriptional factors must be imported to the nucleus for transcriptional processes, while the export of mRNA and ribosomal subunits are also required for cytosolic translation [40]. Many viruses directly interact with components of the nuclear pore complex (NPC), such as nucleoporins, to enable full viral entry into the nucleus, genome import, and the evasion of immune defenses [41]. In contrast, bacterial pathogens typically rely on secreted effector proteins to disrupt nuclear transport and reprogram host transcription. Despite these differences, both viral and bacterial strategies converge on targeting nuclear transport and chromatin-associated processes to establish infection [42].

The ongoing arms race between host and pathogens has driven the rapid evolutionary adaptation of effector proteins, specialized proteins that reprogram host cellular functions, and the unconventional secretion pathways that deliver them into host cells [43,44]. Previously, effector proteins that induce epigenetic alterations without changing the DNA sequence were termed epigenetors, emphasizing their role in reshaping host gene expression through chromatin-based mechanisms [45]. More recently, several bacterial effector proteins operating within the host nucleus have been clearly identified, which has led to the definition of a distinct class of effectors, known as nucleomodulins [46,47].

Nucleomodulins have evolved to mimic host nuclear proteins by harboring eukaryotic-like nuclear localization signals (NLSs), which are short peptide motifs that facilitate nuclear directioning. This closely resembles the strategies employed by host nuclear-directed proteins [48]. Nucleomodulin NLSs enable host nuclear import machinery activity, and experimental data have demonstrated that these effectors are translocated into the nucleus. Here they modulate key nuclear processes, including chromatin organization, histone modification, DNA methylation, RNA splicing, DNA replication and cell cycle control, redirecting the host cellular environment [49,50,51]. All those mechanisms, including epigenetic rewiring consequences, help the pathogen to establish a compatible niche for survival, exploiting the host for replication and dissemination.

**Figure 1:**
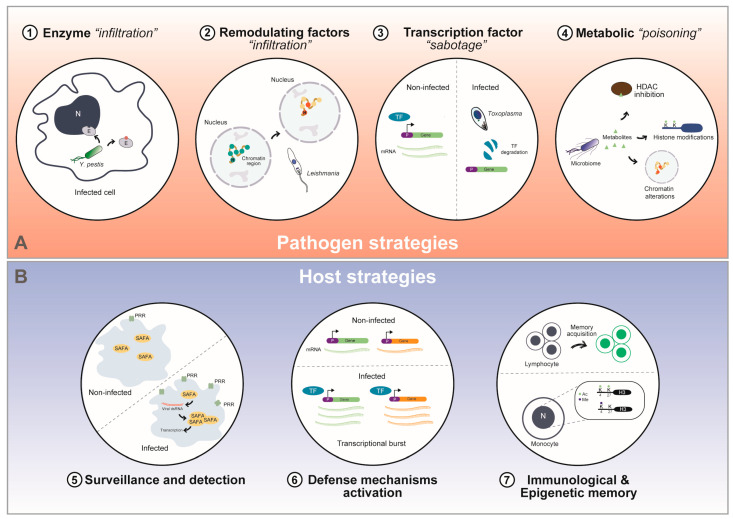
Schematic representation of pathogen and host strategies during infection. (**A**). Major direct and indirect mechanisms for pathogens to attack chromatin. (1) Direct example of pathogenic enzyme secretion after infection. (2) Release and infiltration of pathogen’s chromatin-remodulating factors. (3) Subversion and sabotage done to transcriptional factors, such as NF-κB. (4) Indirect metabolic poisoning of host epigenetics due to metabolite production by pathogens. (**B**). Major examples of host responses during infection. (5) Up-regulation of host’s surveillance, such as SAFA, and pattern-recognition detection mechanisms; (6) Transcriptional burst and/or synthesis of defense proteins after infection; (7) Immunological and epigenetic memory acquisition as a consequence of infection. Nucleus (N); effector enzyme (E); histone (H); transcription factor (TF); promoter (P); lysine at histone-tails (K); pattern-recognition receptor (PRR); acetylation (Ac) and methylation (Me).

Actively trying to survive and subvert the system, the pathogens generally interfere with the host chromatin using a variety of mechanisms, such as the secretion of epigenetic effectors, degradation or repurposing of host enzymes, modulation of signaling pathways, and metabolite production. Most of these can be categorized as direct or indirect strategies that attack the host’s transcriptional regulatory capacities. Simultaneously, host cells attempt to defend themselves and counterattack via the rapid detection of pathogens, the recruitment of chromatin-binding proteins to inflammatory gene loci, transient acetylation bursts at gene enhancers, activation of antimicrobial peptides and more. All those mechanisms are visually illustrated in Figure 1. This constant tug-of-war defines the epigenetic battleground reviewed in this work.

### 2.1. Direct Strategies to Attack Host Chromatin

When the host comes into contact with pathogens, the epigenetic battle begins. Pathogens actively try to divert host gene expression, turning the epigenetic balance in their favor and the chromatin into a conflict zone between defense mechanisms and attack strategies. Broadly speaking, pathogens deploy several recurring classes of epigenetic-centered strategies, frequently involving the direct secretion or injection of effectors with intrinsic activity, such as acetyltransferases or methyltransferases, PTM sabotage, or by hijacking host chromatin-binding proteins. Usually, those pathogen strategies directly interfere with host chromatin and PTMs to induce or inhibit HDAC and HMT activity. In this way, they suppress gene transcription due to a chromatin lockdown, or PTM blackout, which leads to immune-gene suppression and the promotion of intracellular persistence [46,52].

Among virus factors, the adenovirus small e1a can derepress hundreds of individual *Alus* retrotransposons by promoting TFIIIB recruitment in primary human fibroblasts after infection. Retrotransposons are usually subject to tight epigenetic silencing. However, epigenome profiling revealed that e1a decreases H3K27 acetylation and increases H3K4 mono-methylation at derepressed *Alus*, making them resemble poised enhancers [53]. Several viruses can transform infected cells into viral factories by encoding proteins that interfere with cellular acetylation dynamics. HATs like CBP/p300 and their signaling pathways are directly affected by these viral proteins, leading to the control of inflammation and innate immune responses, allowing the viruses to evade immune detection and dampen antiviral signaling [54].

A canonical example of bacterial acetylation-based immune suppression is provided by *Yersinia pestis*, which secretes the effector YopJ, an acetyltransferase that strongly inhibits NF-κB signaling [55]. The major virulence mechanism of pathogenic *Yersiniae* is a type III secretion system (T3SS), which pathogenic species rely on to inject *Yersinia* outer proteins (Yops) into immune cells [56]. YopJ acetylates and inhibits key components of both the NF-κB and MAPK pathways, leading to a profound suppression of pro-inflammatory gene expression downstream of Toll-like receptors (TLRs) [55,57]. The YopJ homolog from *Aeromonas salmonicida*, the AopP effector protein, also disrupts NF-κB signaling downstream of IKK complex activation but, unlike YopJ in the *Yersiniae*, does not disrupt the MAPK signaling pathway [58]. Genome-wide chromatin analyses have since confirmed that pathogen-associated molecular patterns (PAMPs) trigger widespread remodeling of histone modifications across thousands of host loci. This effect is further amplified or redirected by bacterial effectors, such as YopJ, with epigenetic acetylation activity [59].

Other Gram-negative bacteria deploy protein effectors with SET-domain-like folds capable of methylating host histones at non-canonical residues or contexts, effectively importing their own “epigenetic engineers” to sabotage the host chromatin architecture [46,60]. These methyltransferase mimics can rapidly impose a transcriptionally silent chromatin state, preventing the activation of interferon-mediated defense programs. SET (Suppressor of Variegation, Enhancer of Zeste, Trithorax) domains are ubiquitous in eukaryotic lysine methyltransferases. However, secreted SET-domain effectors have been identified in obligate bacterial pathogens, such as *Chlamydia trachomatis*, *Bacillus anthracis*, and *Legionella pneumophila*. Given the absence of histone substrates within bacteria, these organisms can be thought to have co-opted SET-like domains specifically to target host chromatin, thus conferring methyltransferase activity to those bacteria [61].

*Mycobacterium tuberculosis* exemplifies another pathogen that mounts a multi-layered assault on host epigenetic regulation for its survival, inducing extensive nucleosome repositioning and altered histone acetylation at immune gene loci, to limit antibacterial responses during chronic infection [62,63]. The *M. tuberculosis* (*Mtb*) genome is unusually enriched in enzymes with post-translational modifying capacity. This genomic bias is partnered with the secretion of numerous enzymatic effectors, particularly methyltransferases, which constitute over half of the predicted modifying enzymes. This points to a central role for methylation in *Mtb* biology and host interaction. *Mtb* secretes the methyltransferase Rv2067c into macrophages, where it directly tri-methylates histone H3 at lysine 79 (H3K79me3) in a non-nucleosomal context [64]. The Rv2067c-mediated deposition of H3K79me3 at genetic loci such as *TMTC1*, *SESTRIN3*, and *NLRC3* also enhances their expression and facilitates the evasion of host immune responses. In parallel, another secreted methyltransferase, Rv1988, di-methylates arginine 42 within the histone H3 core (H3R42me2), a residue positioned at DNA entry–exit points of the nucleosome. It thereby alters nucleosome dynamics and represses genes involved in reactive oxygen species production (*NOX1*, *NOX4*, *NOS2*) and immune signaling (*TRAF3*, *TNFAIP2*) [65]. Additional effectors further expand the *Mtb* repertoire: the enhanced intracellular survival protein (EIS) promotes IL-10 expression via H3 acetylation, while Rv3423.1 directly acetylates H3K9/K14 after associating with host genome. This likely occurs by binding to host chromatin, as was seen in the isolated chromatin of *Mtb*-infected THP-1 macrophages [66]. Collectively, *M. tuberculosis* effectors drive the accumulation of repressive epigenetic marks such as H3K9me2 and H3K27me3 in immune promoters, while simultaneously redirecting host acetylation activity to silence defense genes.

Fungal pathogens can also deploy direct epigenetic toxins. The HC toxin produced by *Cochliobolus carbonum* acts as a potent inhibitor of histone deacetylases in maize, promoting infection by globally increasing histone acetylation [67,68].

Rather than bringing their own enzymatic weapons, some pathogens instead directly hijack the host’s endogenous machinery. Pathogen proteins may function as decoys or scaffolds that recruit chromatin-complexing proteins to specific genomic locations, enabling broad transcriptional repression or selective pathway activation that favors the pathogen.

Among viruses, adenoviruses provide a striking example of chromatin mimicry. They encode protein VII, a highly basic protein that resembles cellular histones [69]. Protein VII associates with host nucleosomes and limits DNA accessibility by retaining members of the high-mobility group B (HMGB) protein family—including HMGB1, HMGB2, and HMGB3—within chromatin, thereby constraining host transcriptional defense responses [70]. Other viruses, such as HIV, can manipulate host histone acetylation through interactions with p300/CBP to reprogram host chromatin states [71]. Proteomic analyses further reveal that both RSV and HIV-1 Gag proteins can be extracted from euchromatin and heterochromatin fractions, strongly interacting with multiple histones (H1, H2A, H2B, H3, and H4), such as class II HDACs (including HDAC4 and HDAC6). They also interact with seven components of the ATP-dependent chromatin remodeling SWItch/Sucrose Non-Fermentable (SWI/SNF) complex as binding partners [72,73]. Similarly, the Epstein–Barr virus tegument protein BKRF4 binds to histone H2A–H2B dimers via a histone chaperone-like motif. Its distinctive “triple-anchor” binding mode with DWP motif confers unusually strong H2A–H2B affinity, enabling chromatin mimicry that modulates host chromatin organization and the DNA damage response (DDR) [74].

Among Gram-positive bacteria, *Listeria monocytogenes* employs the effector LntA, which enters the nucleus and antagonizes the chromatin repressor BAHD1. LntA is a small basic protein organized into five alpha helices with a putative NLS in its central part [75]. BAHD1 normally compacts chromatin into heterochromatin through interactions with heterochromatin proteins (HP1), histone methyltransferases such as G9a, and the deacetylases HDAC1/2, leading to transcriptional repression [76]. By interfering with this complex, LntA indirectly reshapes chromatin structure and the acetylation states in type I interferon-regulated genes [77].

Other intracellular bacteria deploy nuclear-targeting effectors with enhancer-level specificity. *Ehrlichia chaffeensis* expresses TRP47, its most highly transcribed gene during mammalian infection. TRP47 enters the host nucleus via an MYND-binding domain-dependent mechanism and predominantly binds enhancer regions of genes involved in signal transduction, cytoskeletal organization, immune responses, cell proliferation, and apoptosis. TRP47-bound genes also include RNA-coding genes, many of which are linked to cell proliferation or apoptosis [78].

Finally, protozoan parasites also exploit chromatin dynamics and histone mimicry. Intriguingly, proteomic analyses of *Leishmania donovani* secretome reveal abundant histones among the secreted proteins [79]. Secreted and ectopically produced *Leishmania* histone H3 (LmaH3) can be incorporated into human chromatin, forming nucleosomes with human H2A, H2B, and H4. Structural analyses using crystallography demonstrate that LmaH3-containing nucleosomes are less stable, display weakened H3–H4 interactions, and resist Mg^2+^-mediated chromatin compaction [80]. These altered biophysical properties support the model in which parasite-derived histone effectors directly modulate host chromatin architecture during infection.

### 2.2. Indirect Strategies to Attack Host Chromatin

In addition to direct enzymatic attacks on chromatin and hijacking host machinery, many pathogens can reshape the host epigenetic landscape indirectly by rewiring how transcription works, interfering with intracellular signaling pathways and the host metabolic circuits. Across diverse pathogens, the strategic objectives usually converge to prolong the intracellular survival environment, silence antimicrobial programs, and shape an immune response that favors persistence.

Most viruses have a relatively short replication cycle and kill cells rapidly. Many also induce a phenomenon termed “host shut-off” by globally destabilizing host mRNAs to favor viral protein synthesis [81]. For example, during lytic infection, herpes simplex virus type 1 (HSV-1) induces a dramatic host-to-virus transcriptional switch accompanied by large-scale chromatin condensation, driven by the hijacking of host RNA polymerase II and topoisomerase I [82,83,84].

Kaposi’s sarcoma-associated herpesvirus (KSHV) extends this strategy as its genome circularizes into an episomal mini-chromosome during infection [85], epigenetically hiding most viral genes through methylation and histone modifications, and only allowing specific latent genes to remain active during the latent state. Reactivation from latency also requires chromatin remodeling, including the removal of repressive marks and increased accessibility to both viral and host transcription, thereby recovering genes necessary for the lytic replication [86]. The viral latency-associated nuclear antigen (LANA) orchestrates these changes by recruiting host epigenetic tools, including the H3K4me3-methyltransferase hSET1 and the H3K9me1/2-demethylase KDM3A. The transition between euchromatin and heterochromatin on the KSHV episome is governed by Polycomb repressive complexes PRC1 and PRC2, which are actively recruited by LANA to suppress lytic gene expression following de novo infection [87,88].

Among bacteria, *Salmonella enterica* exemplifies extensive signaling sabotage. The ability of this pathogen to stimulate intestinal inflammation also depends on the protein-delivery machinery T3SS. This system “injects” bacterial effector protein into host cells to modulate a variety of cellular functions for the pathogen’s benefit [89]. This pathogen has a highly diverse effector arsenal to reshape signaling pathways and transcriptional outcomes. It can inject over 40 effector proteins into host cells, with at least 25 of them mediating eukaryotic-like biochemical PTMs of host proteins [90]. The effector repertoire includes both enzymatic modifiers and adaptor proteins that co-opt host enzymes. For example, the tissue-specific expression levels and timing of SPI-1 effector protein fine-tune infection outcomes. In addition, AvrA protein, a multifunctional effector, suppresses NF-κB activation and apoptosis through the inhibition of JNK signaling [91,92]. In contrast, secreted SopA effectors promote inflammation by targeting host E3 ubiquitin ligases TRIM56 and TRIM65, enhancing interferon-β production through RIG-I and MDA5 signaling [93,94]. These examples underscore how *Salmonella* dynamically balances inflammatory and anti-inflammatory programs via indirect chromatin-linked signaling modulation and transcriptional regulation.

*Listeria monocytogenes* also provides a well-characterized example of indirect signaling-mediated histone and chromatin modification. Engagement of the surface receptor Met by the bacterial protein InlB activates PI3K/AKT signaling, leading to the deacetylation of histone H3K18 and widespread transcriptional reprogramming [95]. Subsequent studies have revealed that the disruption of the SIRT2–TDP-43–R-loop signaling axis results in excessive DNA damage during infection, reduced host cell viability, and restriction of *Listeria* infection, emphasizing the importance of host deacetylation pathways for bacterial survival [96]. More broadly, *L. monocytogenes* recruit deacetylase-containing complexes to host promoters, reducing acetylation at IFN-related genes and suppressing antibacterial transcriptional responses [97]. Similarly, the virulence factor OrfX from *Listeria* targets the nucleus and interacts with RybP, a zinc-finger adaptor protein associated with Polycomb-repressive complexes, thereby restricting oxidative responses [98].

Indirect nuclear regulators are targeted by other bacterial pathogens. *Francisella tularensis* secretes virulence factors such as IglJ and VgrG into macrophages during infection. This can interfere with the Apoptosis-Inducing Factor (AIF), contributing to host reprogramming, as was observed with live vaccine strain (LVS) during infection into macrophages [99]. *Vibrio parahaemolyticus*, the leading cause of bacterial seafood-borne diarrheal disease, deploys the T3SS effector VgpA, which localizes to the host nucleolus and binds Epstein–Barr virus nuclear antigen 1-binding protein 2 (EBP2). This interaction shifts c-Myc to the nucleolus, increases rRNA transcription, and promotes host cell proliferation, facilitating gut barrier disruption and bacterial colonization [100].

Protozoan parasites can also exploit indirect epigenetic reprogramming via proteolysis and by triggering host histone modification. *Leishmania* infection indirectly modulates host immune transcriptomes by the proteolysis of transcription factors, such as AP-1 and NF-κB, substantially leading to immune evasion because of the recruitment and activation of immune cells [101]. Altering HDAC activities could result in regulated cytokine production, antigen presentation, and oxidative bursts. In fact, in infected THP-1 macrophages, HDAC1 occupancy increases at defense gene promoters, leading to reduced H3 acetylation and a downregulation of mRNA expression of defensins and antimicrobial genes [102,103]. Comparably, suppression of inducible NO synthase (iNOS) in an HDAC1-dependent manner has also been observed during *Leishmania amazonensis* infection, suggesting an immune system evasion mechanism that is the result of PTM activity modulation [104]. Putative effector molecules secreted by *Leishmania* could modulate host immune transcriptome by altering host epigenome, their crosstalk, and downstream consequences. In *L. donovani* infection, host histone modifiers, such as methyltransferases Ash1l, Smyd2, Ezh2 and demethylases like Kdm5b and Kdm6b, are increasingly expressed. This reshapes H3K4, H3K27, and H3K36 methylation at promoters of *TNF-α*, *IL-12*, *iNOS*, *arginase-1*, and *IL-10*, thereby redirecting macrophage polarization toward a permissive state [105]. Chromatin immunoprecipitation analysis has revealed that *L. donovani* facilitates H3K36 di-methylation at TNF-α promoter by Smyd2 and H3K27 tri-methylation at iNOS promoter by Ezh2, thus suppressing their expression in macrophages. In addition, infection-induced demethylases modulated H3K4 and H3K27 tri-methylation at the IL-12, TNF-α, and arginase-1 promoters. In contrast, Ash1l induced the expression of H3K4 tri-methylation at the IL-10 promoter [105]. All these mechanisms strengthen the powerful immune subversion employed by *Leishmania* and reinforce the central role of host deacetylases in parasite immune evasion [106].

Finally, indirect chromatin modulation can arise from microbial metabolism itself. Microbial metabolites such as short-chain fatty acids (SCFAs), particularly butyrate, act as a context-dependent acetylation inhibitor, depending on concentration and tissue niche, and are abundant in polymicrobial and mucosal environments [107,108]. This *n*-butyrate SCFA produced by commensal gut bacteria as a metabolite by-product is a potent HDAC inhibitor and can also repress LPS-inducible inflammatory responses [109]. Experimental evidence in the intestine has shown that butyrate downregulates LPS responses and contributes to immune homeostasis by modulating macrophage function [110]. Certain oral pathogens also produce butyrate, suppressing H3 acetylation at key cytokine promoters while selectively inducing others, leading to chronic inflammation [97,111].

Metabolic constraints further modulate this balance. The availability of key cofactors for chromatin-modifying enzymes, such as acetyl-CoA, NAD^+^, SAM, and α-ketoglutarate, directly influences acetylation dynamics and can bias the epigenetic state towards immune outcomes of activation or suppression [112,113]. Overall, the examples presented here, as direct and indirect mechanisms, may conceptually overlap in diverse situations. For example, a directly secreted acetyltransferase may have indirect effects by targeting non-nuclear proteins. Alternatively, an indirect metabolic “poisoning” of the cells may lead to a direct lack of essential substrate for histone acetylation. Nevertheless, these direct and indirect strategies collectively position chromatin as an actively contested environment, where pathogens employ layered offensives strategies (enzymatic, signaling, and metabolic) to breach epigenetic defenses and reshape host transcriptional sovereignty.

### 2.3. Correlational Pathogen-Driven Chromatin Remodeling

A common consequence of infection is the pathogen-driven repression or redistribution of activating histone acetylation marks, often resulting in the suppression of immune gene expression. Numerous studies have also demonstrated strong correlations between infection and global changes in chromatin organization, histone modifications, and transcriptional programs. This occurs even when pathogens do not directly modify histones and the epigenetics themselves [114]. However, the extent to which some observed chromatin changes represent adaptive host responses versus direct or indirect pathogen-driven subversion remains to be fully resolved, highlighting a need for deeper mechanistic dissection.

For example, viral infections provide compelling data of signaling-driven chromatin remodeling. In HIV-infected individuals, circulating neutrophils display elevated levels of H3K4 tri-methylation (H3K4me3) and widespread transcriptional dysregulation. These chromatin architecture alterations correlate with impaired activation of the canonical NF-κB pathway, contributing to neutrophil dysfunction and compromised innate immunity [115]. Coronaviruses similarly exploit epigenetic routes to suppress antiviral defenses while the immune system works to defend itself. SARS-CoV-2 infection alters specific host DNA methylation patterns at loci such as *ACE2* and interferon (IFN)-related genes and histone epigenetic modifications in airway epithelial and resident immune cells. This impacts virus entry, replication efficiency, antigen presentation, immune responses (innate and adaptive) and disease severity [116,117].

During acute *Brucella* infection, host cells undergo extensive chromatin reprogramming as part of the defensive response and the subversion attempts of the bacteria. Using integrated data from Hi-C, ATAC-seq, and RNA-seq to generate multi-omic datasets, Xie et al. showed up-regulation of *Tnf* and *Gbp* genes. This enhances antibacterial activity through the disruption of bacterial membrane integrity or activation of inflammasomes. This also leads to the induction of chemokines to promote immune cell recruitment and the suppression of host cell proliferation to limit bacterial replication. Modulation of cell cycle regulators, such as *Ccna2* and *Ccnb2*, reinforces the arrest of cell cycle progression by the host [118]. Such multi-omic approaches exemplify how correlational chromatin signatures can be linked to functional immune outcomes when combined with signaling pathway analysis.

*Trypanosoma cruzi* infection induces extensive transcriptional remodeling in cells, including up-regulation of histone and ribonucleoprotein genes. This suggests that chromatin reorganization and DNA accessibility may influence immune tolerance and parasite persistence. *T. cruzi* infection also recruits DNA damage response proteins to host chromatin, alters chromatin remodeling enzymes, and induces oxidative stress-mediated DNA lesions, a state that appears to favor parasite replication at substantial cost to the host genome [119]. In addition, a downregulation of apoptotic genes 72 h post-infection was observed in trophoblastic cells, further suggesting that *T. cruzi* manipulates host cellular processes to evade immune defenses [120]. Notably, *T. cruzi* migrates toward the host nucleus during its intracellular cycle, indicating a possible interaction with nuclear processes. Although the functional significance of this localization remains unclear, evidence points to disruption of host metabolic pathways and nuclear organization [121].

To bring correlational data to a more rigorous mechanistic understanding, experimental strategies can combine epigenomic profiling, such as single-cell approaches [122], with targeted perturbations (usually as deletion mutants in pathogens, chemical inhibition of chromatin-related enzymes, or temporal infection models). These can help to establish the causal links between pathogen activity, chromatin remodeling, and transcriptional/immunological outcomes.

The following section shifts from strategies to case observation, capturing snapshots of the epigenetic battlefield that reveal who acts, as well as where, when, and how the pathogenic fronts advance in processes within the chromatin landscape.

## 3. Snapshots from the Battlefield: Pathogenic Fronts

The strategy convergence of viral, bacterial and parasitic pathogens on exploiting physiological chromatin processes underscores the universality of epigenetic conflict: in nearly every intracellular infection studied, the earliest and most decisive blows on host defenses usually involve attacks on chromatin-modifying systems, which sets the balance between transcriptional openness and repression. Understanding these attacks will help to clarify how pathogens evade immunity and offer possible therapeutic opportunities to reinforce the host’s chromatin defenses. Due to the diverse amount of literature available, emphasis will be placed on single case studies of some of the most paradigmatic examples of host–pathogen epigenetic interactions.

### 3.1. Virus: Influenza

Viral infection provides a biological context in which two different genomes co-exist and can interact. As viruses have co-evolved with their hosts, they have developed sophisticated strategies to evade, suppress, or reprogram antiviral defenses. Rather than simply altering the host genetic sequence directly, most successful viral infection requires extensive reprogramming of host gene expression and epigenome to balance viral replication against host immune activation. This coexistence ecology provides a powerful model for uncovering fundamental principles of genome organization, chromatin architecture, and how perturbations in genome folding and nuclear topology influence gene regulation [84,123].

A well-characterized example of viral epigenetic interference is provided by the influenza A virus. The nonstructural protein NS1 from influenza A employs a striking form of histone mimicry: its carboxy-terminal region shares sequence homology with the amino-terminal tail of histone H3, allowing NS1 to interact with transcription machinery that normally recognizes the H3K4 mark to initiate transcription. In the H3N2 influenza A virus, this mimicry directly interferes with epigenetic control of host gene expression and suppresses the initiation of innate immune responses [124].

The Influenza NS1 can disrupt chromodomain-associated histone methylation “reader” proteins at interferon-stimulated genes, thereby attenuating antiviral transcriptional responses [125]. NS1 interference with chromatin function may complement other suppressive functions of the influenza virus and provide it with a tool to affect host gene expression in a highly selective fashion by recognizing and using epigenetic patterns of the host [126]. This NS1 from the influenza virus exemplifies a tactic in which pathogen effectors can jam the host’s signal-boosting machinery and calls for reinforcement.

While the viruses manipulate the host cell for its own gain, the host strives to simultaneously contain infection and restore homeostasis. This ongoing molecular competition drives rapid and often reversible epigenetic changes within the host cell [127].

### 3.2. Bacteria: Legionella

Chromatin modifications, whether directly occurring on histone proteins or indirectly on cellular pathways, are frequent targets during bacteria–host crosstalk. A prime example comes from the Deep-branching Intracellular Gammaproteobacteria (DIG), which are among the oldest groups of professional bacteria endosymbionts. They are found all over the world and have specific molecular mechanisms that enable them to interact with their different hosts in varied environments and mutualism–parasitism states. DIGs are classified within the *Legionellales* order, which is traditionally divided into two families, namely *Legionellaceae* and *Coxiellaceae*, along with several related intracellular groups [128].

*Legionella pneumophila* represents one of the most sophisticated and instructive models of host epigenetic manipulation among bacterial pathogens. *L. pneumophila* is a Gram-negative environmental bacterium that persists planktonically in aquatic ecosystems, colonizes biofilms, and naturally infects a multitude of hosts. Upon inhalation of *Legionella*-contaminated aerosols, it acts as an opportunistic human pathogen, replicating within alveolar macrophages, causing a severe type of pneumonia known as Legionnaires’ disease [129].

A defining feature of the biology of *L. pneumophila* is its extraordinarily broad host range among unicellular eukaryotes, spanning multiple evolutionary lineages. The bacterium can replicate intracellularly in ciliated protozoa, in numerous amoebae species such as *Acanthamoeba*, *Echinamoeba*, and *Naegleria*, and in humans [130]. This ecological versatility has driven the evolution of highly adaptable strategies that allow *Legionella* to hijack host proteostasis and promote protein degradation. This powers its intracellular replication in a diverse range of amoebal hosts, as well as in the evolutionary distant human host. No other generalist pathogen microbe has such a broad host range or extensive protein effector redundancy and diversification, and no other pathogen replicates within protists as well as *L. pneumophila* [131].

Central to *L. pneumophila* pathogenesis is the intracellular multiplication/defective organelle trafficking (Icm/Dot) type IV secretion system (T4SS). This system injects more than 300 effector proteins into eukaryotic host cells to govern interactions within those cells, promotes formation of the *Legionella*-containing vacuole (LCV) and subverts cellular processes in favor of the pathogen [132,133]. The combination of genome plasticity, extensive effector diversity, and the broad host range across the *Legionella* genus also establishes it as an archetype system for studying pathogen evolution, functional genomics, chromatin manipulation, and host–pathogen co-adaptation [134].

Among these hundreds of effectors are chromatin-targeting enzymes that directly manipulate host histone modifications. The effector RomA is a eukaryotic-like SET-domain histone methyltransferase that tri-methylates histone H3 at lysine 14 (H3K14me3), counteracting host immune gene expression. In *L. pneumophila*, structural analysis of the effector RomA in the complex with a histone H3 peptide revealed that its C-terminal ankyrin repeats play an essential role in binding the H3 tail. Ankyrin-repeat proteins are versatile mediators of protein–protein interactions and are frequently co-opted by obligate intracellular bacteria to manipulate host cellular processes. The loss of these ankyrin domains abolishes RomA histone methyltransferase activity, demonstrating that ankyrin-mediated chromatin engagement is required for enzymatic modification of host histones [135,136]. RomA acts in parallel with LphD, another secreted effector enzyme with histone deacetylase activity that specifically removes H3K14ac in synergy with RomA. Both effectors converge on the HBO1 histone acetyltransferase complex, which normally acetylates H3K14, thereby enforcing a repressive chromatin state at immune-related loci [137]. These findings support the molecular basis by which a conserved bacterial histone methyltransferase or acetyltransferase is injected into the host cells and directly targets nuclear chromatin to reprogram gene expression.

*Legionella* remains the most prolific human pathogen to replicate within various unicellular eukaryotic hosts. While the basic biology of macrophages and that of phagocytic protists are thought to be similar enough for intracellular replication of *L. pneumophila*, there are major notable differences between the two evolutionarily distant phagocytic host cells upon injection by the bacteria. In macrophages, the bacterium actively suppresses apoptosis by triggering both NF-κB-dependent and independent anti-apoptotic pathways, thereby prolonging host cell survival to support bacterial replication [138]. At the epigenetic level, *L. pneumophila*-derived factors reprogram infected macrophages toward a tolerogenic state characterized by increased H3K9me3 and reduced H3K4me3. These chromatin changes are associated with persistent heterochromatin organization, correlated with decreased gene transcription [139]. In parallel, infection induces substantial up-regulation of HDAC6 expression in murine lung tissue. While HDAC6 participates in phagosome–lysosome fusion and autophagy-mediated bacterial clearance, its precise contribution to *Legionella* invasion and persistence in macrophages remains unclear [140].

*L. pneumophila* effectors also encode “effectors of effectors”, a subset with the function of modifying activities of other effectors inside the host. For example, upon activation by host calmodulin, SidJ glutamylates and inhibits the ubiquitin ligase activity of SidE effectors, preventing excessive ubiquitination that could disrupt host ubiquitin homeostasis [141,142]. Often identified through experimental serendipity while studying classical effectors, these “metaeffectors” add a new layer to *Legionella* parasitism repertoire and further demonstrate their effector diversity [143,144].

### 3.3. Fungi: Phytophtora

Fungal and oomycete pathogens are now known to modulate host HAT and HDAC expression as a strategy to repress immune genes, which shows how shifts in chromatin landscapes can directly shape disease susceptibility across multiple systems. Host–pathogen epigenetic interactions are also a relevant topic in plants and crops research, with *Phytophthora* species occupying a particularly prominent position among plant-infecting microbes. Derived from Greek for ‘plant destroyer’, these very diverse fungus-like microbes cause diseases in various species of plant, including many of major agricultural importance [145,146]. Their success as pathogens is closely linked to their ability to manipulate host epigenetic regulation, especially histone acetylation, to suppress plant immunity and promote the disease.

A striking example is provided by PsAvh52, an early-induced effector with the Arg-any amino acid-Arg-Leu (RxLR) domain that is secreted by *Phytophthora sojae*, the causative agent of soybean root rot. PsAvh52 is required for full virulence and suppresses host immune responses by targeting, in vivo and in vitro, a previously uncharacterized soybean transacetylase, GmTAP1. Upon interaction, PsAvh52 induces the translocation of GmTAP1 from the cytoplasm into the nucleus, where it acetylates histones H2A and H3. This results in a global increase in H2AK5 and H3K9 acetylation during early infection, thereby reshaping chromatin accessibility and promoting host susceptibility to *P. sojae* [147,148].

*Phytophthora* employs additional, mechanistically distinct strategies to interfere with host histone acetylation. The cytoplasmic effector PsAvh23 acts as a modulator of HATs in plants by disrupting the function of the Spt-Ada-Gcn5 acetyltransferase (SAGA) histone HAT complex, as it binds with its ADA2 subunit and prevents its association with the catalytic GCN5 [149]. Although there has been some progress, major challenges remain, particularly in elucidating the regulatory mechanisms of acetylation. In addition, little is known about the mechanism that histone epigenetic uses to modulate plant resistance, about fungal pathogenicity, or how histone marks interact with other epigenetic modifications to orchestrate plant–pathogen dynamics [150].

### 3.4. Protozoa: Toxoplasma

*Toxoplasma gondii* employs a highly sophisticated, multilayered and multi-effector strategy to manipulate host cell functions. This is achieved through the secretion of numerous protein effectors that originate from rhoptries and dense granules organelles into the host cell. While rhoptry proteins are delivered during the early stages of invasion, immediately after parasite attachment, dense granule proteins (GRAs) are usually secreted after the parasite has successfully entered the parasitophorous vacuole, enabling sustained modulation of host signaling and transcriptional programs [151,152].

A central target of *T. gondii* manipulation is the interferon-γ (IFN-γ) signaling axis, the cornerstone of cell-autonomous immunity. IFN-γ regulates the expression of more than 1000 genes involved in cell-autonomous immune defense, controlling immune responses and immune-independent processes [153]. Upon receptor engagement, IFN-γ activates signal transducer and activator of transcription (STAT1), which translocates to the nucleus and binds gamma-activated sequence (GAS) motifs in the promoters of IFN-γ responsive genes [154]. *T. gondii* subverts these interferon responses pathways by manipulating epigenetic regulation through secreted effectors: the dense granule effector TgIST (*Toxoplasma* inhibitor of STAT1 transcriptional activity) and the *Toxoplasma* NCoR/SMRT modulator, TgNSM [155,156]. Acting cooperatively, both these effectors repress IFN-γ-driven transcription by recruiting the host co-repressor complexes to STAT1-bound promoters. This blocks the expression of key immune genes, including PKR and MLKL, and prevents necroptotic cell death [157]. Parasites lacking TgIST fail to suppress IFN-γ responses in vitro and are avirulent in vivo, underscoring the central importance of this epigenetic interference [158].

*T. gondii* also manipulates host chromatin to control cell cycle progression. The rhoptry effector protein ROP16 targets the epigenetic regulator ubiquitin-like with PHD and RING fingers domains 1 (UHRF1), leading to cell cycle arrest. This is mediated by a DNA methyltransferase (DNMT)-dependent chromatin remodeling at the cyclin B1 gene promoter. The recruitment of phosphorylated UHRF1 to a repressive multienzyme complex induces histone H3 deacetylation and methylation at the promoter, resulting in transcriptional silencing of cyclin B1 and host cell cycle arrest [159]. Together, these strategies illustrate how *T. gondii* exploits epigenetic control points to redundantly and simultaneously suppress immune defenses and reshape host cell fate, which reinforces chromatin regulation as a central interface in protozoan pathogenesis.

### 3.5. Helminths: Nematode Cysts

Cyst nematodes are obligate plant-parasitic helminths that establish long-term feeding structures with their hosts by secreting a complex mixture of effector proteins into plant root cells. These effectors reprogram host gene expression and cellular architecture to generate specialized feeding sites, termed syncytia, which sustain nematode growth and reproduction [160,161]. A growing body of evidence indicates that this reprogramming relies on the manipulation of host chromatin and post-translational regulatory machinery too.

One of the best-characterized examples in this scenario is the sugar beet cyst nematode, *Heterodera schachtii*. This nematode produces the effector protein 32E03, which localizes to the plant nucleus and nucleolus, where it strongly inhibits the activity of multiple *Arabidopsis thaliana* histone deacetylases, including the plant-specific HDAC HDT1 [162]. As a result of the expression of 32E03 in plant cells, increased acetylation of histone H3 across rDNA chromatin is observed for bidirectional regulatory purposes of rRNA gene expression. At low 32E03 effector expression levels, it derepresses a subset of rRNA genes, which are conducive to *H. schachtii* parasitism, enhancing ribosomal output and creating a metabolic environment favorable for nematode colonization. In contrast, high levels of 32E03 induce aberrant bidirectional transcription at rDNA loci, triggering the production of rDNA-derived small RNAs and subsequent RNA-directed DNA methylation, ultimately silencing rRNA genes and impairing nematode development. 32E03 also interacts directly with the *Arabidopsis* FK506-binding protein FKBP53 together with the HDT1 within the nucleolus. However, there are likely to be additional, so far unknown, consequences of 32E03-mediated inhibition of histone deacetylases [162].

*H. schachtii* also exploits another host post-translational signaling machinery. The effector 10A07 accumulates in the cytoplasm of *A. thaliana* cells, where it physically associates with a host kinase and undergoes phosphorylation. This host-mediated PTM serves as a molecular switch that promotes the subsequent nuclear translocation of effector 10A07. When the protein effector 10A07 associates with the auxin-responsive transcription factor IAA16, it alters auxin signaling at the syncytia feeding site. However, in this case, the precise mechanisms by which 10A07 co-opts host post-translational machinery remain unclear [163,164].

Collectively, these findings highlight cyst nematodes as potent manipulators of host epigenetic regulation, demonstrating that helminths effectors can fine-tune chromatin states to balance plant host cell reprogramming with successful parasite development. This further illustrates histone acetylation and related chromatin-based processes act as conserved and exploitable nodes across diverse host–pathogen interactions, whether they are viruses, bacteria, fungi, protozoa or nematodes.

## 4. Biological Outcomes and Countermeasures

The outcome of any infection is determined by a complex interplay between the host’s defense mechanisms and the pathogen’s virulence strategies. Across viruses, bacteria, fungi, parasites, and plant pathogens, this interplay is increasingly understood to be mediated at the level of chromatin, where epigenetic plasticity enables both rapid immune activation and pathogen-driven repression. Briefly, the consequences of an infection usually include pathogen detection and elimination. However, survival and chronic infection may occur, together with immune evasion or memory. Epigenetic scars, modulation of defense gene expression and overall reprogramming of cellular behavior occurs as a result of these chromatin interactions.

### 4.1. Functional Consequences at the Raided Side

Although they originate from different kingdoms, invasive microbes can all trigger an acute primary infectious episode in their hosts, a phase that can vary in duration, intensity, and consequences, but is most often self-limiting in immunocompetent hosts. Nevertheless, some of these microbes, such as *T. gondii*, can persist in dormant stages for a long time in their hosts, silencing the host defenses and overcoming innate and adaptive immunity [165].

On the outcome of viral infection, it has been shown that patients with COVID-19 symptoms exhibit notable signs of increased aging compared to healthy individuals. Genome-wide DNA methylation studies in severe COVID-19 patients suggest accelerated biological aging compared to those with milder symptoms [166].

In general, chromatin changes produce tangible functional consequences that directly influence disease outcomes. One of the best-known mechanisms of chromatin remodeling in response to the pathogen involves the polarization of naive T lymphocytes into CD4 or CD8 subpopulations, which have been previously described [167,168]. In particular, chromatin acetylation at promoters and enhancers of inflammatory genes reshape transcriptional accessibility during infection, leading to a reduced induction of cytokine and interferon-responsive programs, which are critical for early innate defense. In addition, decreased acetylation at MHC class II loci compromises antigen presentation, which limits effective T-cell activation and favors pathogens that rely on chronic or latent infection strategies, such as *M. tuberculosis* [169].

Chromatin remodeling also shapes other immune cell identities. As macrophage is one of the most prime targets of infection, it may dictate disease fate, and the characteristics of parasites may influence the host macrophage plasticity for survival. Macrophages display remarkable epigenetic plasticity [170,171]. Infection-induced chromatin changes can bias macrophages toward pro-inflammatory cytokine-expressing macrophages, which mediate Th1-type responses. Alternatively, they may tend towards the tolerogenic state, with macrophages expressing anti-inflammatory cytokines. Those cells can have different subsets, such as M2a, M2b, M2c, and M2d, which elevate Th2-type responses, thereby influencing whether immune responses clear the pathogen or inadvertently support its persistence [172]. This plasticity extends to epithelial cells and to classical epigenetic marks in chromatin. For example, strain-specific acetylation signatures at H3 (hyper-acetylation at H3K9/H3K14) in *Staphylococcus aureus* mastitis models can influence inflammatory trajectories and determine whether infection becomes acute or chronic [173].

Similar repression of immune responses has been observed in macrophages infected with *L. donovani*, where up to ~40% of host genes show reduced expression linked to epigenomic remodeling [174,175]. Recently, sleepy macrophages that possess a state adaptation to *L. major* infection were identified. These cells boast pro-inflammatory cytokine expression by promoting chromatin remodeling and RNA regulation through transcription factors [176]. Pathogen-induced chromatin remodeling directly shapes disease outcomes, immune polarization, antigen presentation, and cellular differentiation, defining biological consequences of infection and framing the immune countermeasures explored in the following sections.

### 4.2. Host Countermeasures: Activating Immunity

Host cells deploy different strategies and countermeasures as part of innate and adaptive immunity. In an exemplary countermeasure attempt, one of the major defensive approaches that host cells use to protect themselves after viral infection is by local hyperacetylation at the IFN-β promoter, reducing this critical antiviral gene expression [177].

Upon activation of pattern-recognition receptors (PRRs) by Pathogen-Associated Molecular Patterns (PAMPs), rapid and localized increases in H3K27ac and H3K4me3 occur at enhancers and promoters of genes regulated by interferon and NF-κB, which also enables high-amplitude transcriptional bursts during early immune activation [36,178].

The detection of pathogens is mediated by several families of innate PRRs, which include Toll-like receptors (TLRs), RIG-I-like receptors (RLRs), cytosolic double-stranded DNA receptors (CDRs), NOD-like receptors (NLRs), and C-type lectin receptors (CLRs) [179,180]. Other defense proteins help to resolve infections, such as the nuclear scaffold attachment factor A (SAFA), which acts as a chromatin-associated viral dsRNA sensor and trans-activator of antiviral immune genes. After detecting viral RNA, SAFA oligomerizes and aids enhanceosome formation at the distal enhancer of *IFNB1* in a chromatin remodeler-dependent way [181].

Furthermore, at the level of pathogen recognition, chromatin modifiers themselves can function as immune regulators. HDAC6, for example, enhances antiviral immunity by deacetylating the key viral RNA sensor RIG-I at lysine 909, thereby promoting viral RNA recognition and interferon production [182]. Indeed, depletion of HDAC6 expression leads to impaired antiviral responses to RNA viruses, but not against DNA viruses. In general, histone acetylation emerges as a central regulatory node due to its speed, reversibility, and tight coupling to cellular metabolism. This opens the chromatin by depositing acetylation at promoter–enhancer units, thereby allowing the rapid expression of pro-inflammatory genes, making it a hallmark of early immune activation [22,36,114]. Taken together, these chromatin-centered defense strategies enable the rapid activation of immune programs in response to pathogen sensing, while simultaneously establishing regulatory chromatin states that may persist beyond the acute phase of infection.

### 4.3. Aftermath of Infection: Immune Memory and Epigenetic Scars

The phenomenon known as “innate immune memory” or “trained immunity” has forced us to rethink our understanding of how the immune system functions. For a long time, innate immunity was thought to be more primitive and less sophisticated compared to adaptive immunity. However, trained innate immunity can be triggered after a primary stimulation, with increased non-specific response after re-stimulation [183,184]. Epigenetic modifications are at the heart of this cell reprogramming and enable innate immunity cells to display a kind of immunological memory, like that observed in the acquired immunity. In short, the main mechanism by which innate cells develop a memory is through long-term epigenetic reprogramming [185]. It has been previously shown that bacterial nucleomodulins can induce short- or long-term epigenetic modifications of the host cell [49]. It is also worth noting that tolerance and trained immunity are deeply associated with distinct and opposing epigenomic states, which highlights how distinct chromatin states can bias immunity toward protection or restraint [186].

Chromatin can retain residual signatures that extend beyond the acute phase of infection. There is increasing evidence to indicate that pathogen-induced epigenetic alterations in the chromatin landscape may persist after microbial clearance, leaving durable “epigenetic scars” that alter long-term immune tone, disease susceptibility, and the establishment of latency [114,187]. For example, monocytes tolerized by LPS treatments demonstrate H3K4 mono-methylation, as well as a failure to accumulate H3K27 acetylation and other active histone marks at the promoters of tolerized genes, even during a second challenge [188].

While epigenetic regulation enables rapid immune adaptation, it also limits excessive inflammation and collateral tissue damage. However, following severe or chronic infections, this regulatory system may overshoot, leaving adaptive immune cells locked into hypo-responsive states. Within this epigenetic memory context, CD4^+^ T cells may become anergic and CD8^+^ T cells may become exhausted, with chromatin landscapes that overlap those induced by cancer and chronic inflammation [189]. Such scars have been documented following severe viral, bacterial, and parasitic infections. Animal models have demonstrated that post-infectious immune suppression is epigenetically mediated, and that detrimental epigenetic marks induced by chronic infections can also overlap with those induced by cancer [190].

Both sides of this struggle are consistent with the “Red Queen” principle or hypothesis, formulated by Leigh Van Valen in 1973 [191]. Inspired by Lewis Carroll’s literary classic *Alice Through the Looking Glass* from 1871, this concept in evolutionary biology seeks to explain how biological relationships, particularly those involving hosts and parasites, require constant adaptations simply to maintain the status quo in their pathogenic environments. Thus, the host is motivated to evolve epigenetic defense mechanisms to reduce the damage associated with infection and restore homeostasis, while the pathogen evolves attack and evasion mechanisms to overcome the host’s epigenetic defenses. This establishes a cycle that never truly ends, and that drives the continuum evolutionary arms race between both host and pathogen.

## 5. Development of Data Acquisition Strategies

Advances in epigenomics and systems biology are rapidly expanding the experimental and analytical repertoire available to study host–pathogen interactions at chromatin resolution. At the mechanistic level, data from epigenetic regulatory systems, determined by chromatin condensation, nucleosome dynamics, enhancer activation and global accessibility, can be integrated with modern technologies. These parameters are now directly measurable using methods like chromatin immunoprecipitation, ATAC-seq, and single-cell epigenomics, which provides a comprehensive view of transcriptional regulation in living cells [192,193,194].

At the cellular level, single-cell and spatially resolved technologies are transforming how infected and bystander cells are identified and characterized. While most single-cell studies have relied on transcriptomic profiling (single-cell RNA sequencing), single-cell epigenomic approaches, such as single-cell ATAC-seq, offer a promising tool for investigating the epigenomic landscape to discover key components of the regulatory logic that precedes and constrains gene expression [195]. Further advances in single-cell technologies have refined our ability to interrogate chromatin dynamics in complex immune contexts. In 2021, You et al. developed a single-cell approach that integrates T cell receptor sequencing (scTCR-seq), fluorescence-activated cell sorting (FACS) with index sorting and ATAC-seq (termed Ti-ATAC-seq). This enables simultaneous profiling of cell-surface markers, paired TCR sequences, and chromatin accessibility at single-cell resolution. With the application of this strategy, individuals convalescing from COVID-19 were demonstrated to establish immune memory and trained immunity through global remodeling of chromatin accessibility landscapes [196].

Spatial transcriptomics, combined with unbiased detection of host and viral pathogen transcripts, are a new way to identify infected cells, including cells that may not otherwise be marked as infected. In addition, ST data can be used to define the responses of these cells to infection and further subclassify them. Fine details of the spatial–temporal distribution of infected cells, particularly their location relative to vasculature, can also provide new insights into the spread of infection [197]. Spatially resolved assays for chromatin profiling, including the mapping of chromatin accessibility and genomic features, will be useful for studying changes in gene regulation induced by infection. Although challenges remain, including high costs and analytical complexity, the integration of ST with spatial epigenomics, chromatin accessibility, and metabolomics is expected to provide us with a far more comprehensive view of pathogen–host interactions.

Inside the nucleus, chromatin occupies a dynamic three-dimensional nuclear space. Loops and higher-order interactions in this space are essential for maintaining the transcriptional programs that support differentiation, development, and environmental responsiveness [198]. A high-order study of chromatin assembly and organization could also be critical for future research and development [31,199]. Furthermore, it is possible to suggest that the integration of transcriptional and metabolic data in a type of metabolic flux analysis, combined with epigenomic information, may open new frontiers of knowledge, as several PTMs, such as acetylation in the form of acetyl-CoA availability [200], are inherently tied to cellular metabolism.

Additionally, data integration can also extend to dual RNA-seq strategies, which have reshaped the study of intracellular pathogens by enabling the simultaneous profiling of host and pathogen transcriptomes. In *T. cruzi* infections, for example, dual RNA-seq has revealed dynamic host–parasite interactions that are obscured when each organism is studied in isolation. This method captures cell-type-specific susceptibilities and provides a comprehensive view of disease progression and immune responses [201].

Overall, future research may converge on three major objectives: (a) genome-wide mapping of chromatin modifications during infection, such as ChIP-seq profiling of acetylated histones and spatial epigenomics; (b) mechanistic identification and functional characterization of pathogen-derived epigenetic effectors; and (c) testing of epigenetic modulators in physiologically relevant models of infection.

Most conventional therapeutic approaches usually try to target the pathogens that cause disease but given the clear impact of host chromatin in infection, therapies with epigenetic modulators directed at hosts may emerge as attractive countermeasures. Since those epigenetic modifications are reversible, a new frontier of pharmacology is emerging, one that is focused on designing drugs that can transiently modify epigenetics to reduce pathogen access and proliferation [202]. In chronic viral infections, such as HIV, epigenetic therapies are being explored to eliminate persistent reservoirs [203]. HDAC inhibitors have been proposed as promising tools to alter histone acetylation in latent infected CD4^+^ lymphocytes. This would facilitate viral reactivation and potentially purge the reservoir of persistent infection using conventional treatment [204]. However, given the success of current antiretroviral therapy and the relatively good health and life expectancy of patients with suppressed viremia, clinical approaches to eradicate diseases like this must be very safe and tolerable.

We are only beginning to understand the substantial contributions of epigenetics to human diseases, and the full impact on cell and chromatin dynamics remains to be discovered in the complex relationships of host and pathogens. The fine-tuned therapeutic use of epigenetic mechanisms can potentially promote the elimination of resistant pathogens while restraining host immunity response, thus preventing excessive collateral damage to host tissues after severe and chronic infections. Understanding epigenetic patterns using high-resolution epigenomic profiling, while also integrating with innovative pharmacological methods, could transform directed therapies for infectious diseases. This offers new possibilities for reprogramming host chromatin efficiently and limiting pathogen survival. This highlights a growing model in infectious disease biology: restoring host activity states may be as powerful as targeting the pathogen itself.

## 6. Concluding Remarks

The long co-evolutionary history between pathogens and their hosts has provided microbes with remarkable capacities to exploit cellular machinery to survive and replicate. In addition to utilizing host resources, many pathogens act at the level of epigenetic regulation, subtly reprogramming the transcriptional landscapes to favor parasitism while minimizing excessive immune responses. There is a recurring theme across viruses, bacteria, fungi, protozoa and nematode cysts: chromatin is not passive during infection but is an active and highly contested territory for transcriptional control.

Chromatin is the central command headquarters in eukaryotic cells, enabling them to establish defense strategies and mobilize precise and transient immune responses, while simultaneously offering pathogens multiple entry points to overthrow host defenses. The vastly diverse and combinatorially complex interface generated by PTMs, particularly on histones and chromatin-associated proteins, provides both host and pathogen with an exceptionally rich landscape for regulatory strategies. Acetylation, methylation, and other modifications operate in combination on several context-dependent fronts. This allows rapid, reversible, and spatially defined control of chromatin accessibility and gene expression during infection by different types of pathogens.

Understanding how pathogens can manipulate these fundamental axes of transcriptional control is therefore crucial for deciphering the mechanisms of infectious diseases and the environment in which they interact with their hosts. Equally important is to define the stages of epigenetic competition: the kinetics by with each chromatin state is established and erased, the hierarchical integration and correlation of PTMs at different infection contexts, and the precise tools deployed by both host and pathogen to enforce or override transcriptional directives, while in “peace” or at “war”. Progress in these areas should help to clarify how infections are sustained and resolved. It will also inform the rational design of precision disease-directed therapies. By targeting chromatin dynamics and specific PTMs, rather than the pathogen alone, future interventions may help to restore immune competence while avoiding the collateral effects and selective pressures associated with drugs directed at pathogens. This could help to consolidate epigenetic regulation as a central component of next-generation therapies.

## Data Availability

No new data were created or analyzed in this study. Data sharing is not applicable to this article.
